# Dachengqi Decoction: emerging therapeutic potential in sepsis and target organ injury

**DOI:** 10.3389/fphar.2026.1762275

**Published:** 2026-04-28

**Authors:** Guang-Tao Pan, Lin-Rui Ma, Yu Deng, Yu-Han Wang, Zi-Neng Yuan, Lin Zhao, Jing-Yi Yang, Pei-Yu Yan

**Affiliations:** 1 Faculty of Chinese Medicine, Macau University of Science and Technology, Macau, China; 2 Yancheng Hospital of Traditional Chinese Medicine Affiliated to Nanjing University of Chinese Medicine (Yancheng Hospital of Traditional Chinese Medicine), Yancheng, China; 3 Hubei University of Chinese Medicine, Wuhan, Hubei, China; 4 Fudan University, Shanghai, China; 5 Renmin Hospital of Wuhan University, Wuhan, Hubei, China; 6 Faculty of Chinese Medicine, National Key Laboratory of Mechanism and Quality of Chinese Medicine, Macau University of Science and Technology, Macau, China; 7 Zhuhai MUST Science and Technology Research Institute, Zhuhai, China

**Keywords:** Chinese medicine metabolites, Dachengqi Decoction, immune suppression, inflammation, sepsis, target organ injury

## Abstract

Sepsis is a life-threatening systemic inflammatory response syndrome caused by dysregulated host responses to infection, which frequently progresses to multiple organ dysfunction and is associated with high morbidity and mortality. Conventional Western medicine has limited efficacy in controlling inflammation and protecting target organs. In contrast, DCQD, a classical traditional Chinese medicine formula, has attracted increasing attention due to its multi-target pharmacological properties. This review systematically summarize recent advances in the study of DCQD and its bioactive constituents in the treatment of sepsis and sepsis-related organ injuries, and to explore their underlying mechanisms and translational potential. Relevant studies published from 2015 to October 2025 were systematically searched in PubMed, Embase, Google Scholar, Web of Science, CNKI, VIP, and Wanfang databases. Studies focusing on the protective effects and mechanisms of DCQD and its active metabolites in sepsis and target organ protection were included and reviewed. Current evidence indicates that DCQD and its bioactive metabolites exert protective effects against sepsis-induced injury of the gastrointestinal tract, lungs, liver, kidneys, and heart. These protective effects are primarily achieved through multiple mechanisms, including anti-inflammatory, immune regulation, improvement of microcirculation, and attenuation of oxidative stress. DCQD and its active metabolites show significant therapeutic potential for sepsis and associated target organ dysfunction. Their multi-target and multi-pathway mechanisms provide a scientific basis for optimizing treatment strategies and developing novel traditional Chinese medicine-based therapeutics.

## Graphical Abstract

**Figure:**
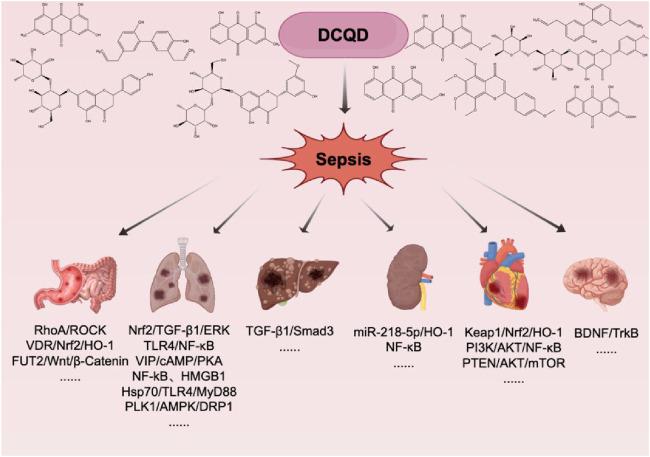
This review summarizes the mechanisms and research progress of Dachengqi Decoction (DCQD) and its active metabolites in the treatment of sepsis and sepsis-induced target organ injury.

## Introduction

Sepsis is a common and life-threatening clinical syndrome in intensive care unit (ICU), characterized by infection-induced immune dysregulation leading to multiple organ dysfunction (Tang et al., 2025). It accounts for approximately 20% of all deaths worldwide (Kim et al., 2024). Conventional treatments mainly rely on antibiotics and supportive care; however, their efficacy remains limited due to excessive inflammation, immune suppression, and insufficient protection of target organs. In recent years, the concept of personalized immunotherapy for sepsis has gained increasing attention, aligning closely with the syndrome differentiation and holistic regulation principles of traditional Chinese medicine (TCM) (Slim et al., 2024). Dachengqi Decoction (DCQD), a classic multi-metabolite TCM formula, has shown promising therapeutic potential in the management of sepsis and its associated target organ injuries, including the gastrointestinal tract, lungs, liver, kidneys, heart, and brain. Its pharmacological effects are primarily attributed to anti-inflammatory activity, immune modulation, and organ protection, which have attracted growing scientific and clinical interest (Yang et al., 2025). However, despite an increasing number of experimental and clinical studies, the mechanistic pathways and translational value of DCQD in sepsis have not yet been systematically summarized. This review aims to provide a comprehensive overview of the research progress and mechanisms of DCQD and its active metabolites in the treatment of sepsis and sepsis-induced organ injury. Furthermore, it evaluates their potential clinical applications, offering new insights for optimizing sepsis management and facilitating the development of novel TCM-based therapeutics. The specific literature retrieval strategy is as follows: First, we manually searched PubMed, Embase, Google Scholar, Web of Science, CNKI, VIP, and Wanfang databases for articles containing “Dachengqi” in their abstracts. We then compiled the active metabolites identified in DCQD into Component A. We searched for Sepsis in combination with Component A(Emodin OR Rheic acid OR Chrysophanic acid OR Magnolol OR Honokiol OR Hesperidin OR Naringin OR Neohesperidin OR Physcion OR Aloe-emodin OR Tangeretin)[Title/Abstract] into Component B. We also searched for Sepsis in combination with Rheum officinale, Zhi shi, Magnolia officinalis, and Mirabilite [Title/Abstract] into Component C. Additionally, we searched for Sepsis in combination with “Dachengqi” [Title/Abstract] into Component D. The final dataset comprised the union of Components B, C, and D. Our retrieval method ensures comprehensive coverage of relevant literature while maintaining high specificity to the research topic. The entire retrieval process is reproducible, with clear inclusion and exclusion criteria established prior to screening. Thus, this study’s retrieval approach aligns with standard scientific methods commonly used in related fields. We consider this retrieval strategy to be scientific, rigorous, and appropriate for this research.

## Pathophysiological mechanisms of sepsis

The pathophysiology of sepsis is highly complex, involving interactions among pathogenic factors, host immune dysregulation, and multi-organ dysfunction. Although its precise mechanisms are still under intensive investigation, significant progress has been made in elucidating its immunopathological processes.

In the early stage of sepsis, pathogen invasion activates the host immune system, triggering an amplified and uncontrolled inflammatory cascade, often referred to as a “cytokine storm” (Tang et al., 2025). This process is characterized by the massive release of pro-inflammatory mediators such as tumor necrosis factor-α (TNF-α), interleukin-1 (IL-1), IL-6, IL-8, and high mobility group box protein 1 (HMGB1), which enter the systemic circulation and initiate systemic inflammatory response syndrome (SIRS) (Jabornisky et al., 2024). The ensuing cytokine storm damages vascular endothelial cells, increases capillary permeability, and disrupts microcirculation, resulting in tissue hypoperfusion and progression to multiple organ dysfunction syndrome (MODS) (Zhang et al., 2023). Moreover, activation of the coagulation cascade and inhibition of fibrinolysis can induce disseminated intravascular coagulation (DIC), exacerbating organ injury and leading to multi-organ failure, which poses a serious threat to patient survival (Cecconi et al., 2018; Vincent, 2022; Yan et al., 2023).

**FIGURE 1:**
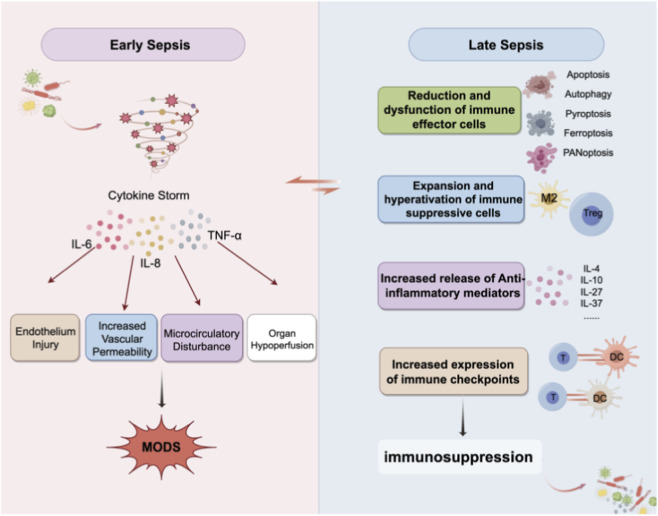
Pathogenesis of sepsis.

In the later stage of sepsis, the host immune homeostasis shifts toward immunosuppression (Liu D. et al., 2022). This phase is characterized by reduced proliferation and differentiation of immune cells, decreased secretion of cytokines and pro-resolving mediators, reprogramming of antigen-presenting cells, and extensive apoptosis of immune effector cells (van der Poll et al., 2017), ultimately leading to immune exhaustion. Such immune paralysis not only impairs inflammation resolution and hinders pathogen clearance but also increases susceptibility to opportunistic infections, aggravating disease progression and contributing to persistent immunosuppression and the development of post-sepsis syndrome (Liu D. et al., 2022; van der Poll et al., 2017; Bode et al., 2023; Venet and Monneret, 2018; Yuan et al., 2022). The pathophysiological mechanisms of sepsis are illustrated in Figure 1.

## Active metabolites and therapeutic potential of DCQD in sepsis

### Research progress of DCQD in sepsis

The pathophysiological mechanisms of sepsis are highly complex and continue to be elucidated. In recent years, TCM has demonstrated considerable potential in the management of sepsis.

Clinical studies (Yu et al., 2010; Yu et al., 2011) have shown that oral administration of DCQD can effectively reduce the levels of inflammatory mediators such as TNF-α, IL-2, IL-6, C-reactive protein (CRP), α1-acid glycoprotein (AAG), and haptoglobin (HP) in patients with sepsis, thereby suppressing systemic inflammation. Moreover, DCQD can modulate lymphocyte subpopulations (CD3^+^, CD4^+^, and CD8^+^), improve immune function, shorten the duration of mechanical ventilation and ICU stay in patients with severe sepsis, and improve Acute Physiology and Chronic Health Evaluation II (APACHE II) and MODS scores, ultimately enhancing the clinical cure rate. Animal experiments have further confirmed that DCQD downregulates IL-1β, IL-6, TNF-α, and myeloperoxidase (MPO) levels, reduces the lung wet-to-dry weight ratio, and alleviates sepsis-induced injury by inhibiting the PI3K/Akt signaling pathway (Fu et al., 2021). In addition, a meta-analysis including 869 patients with sepsis demonstrated that Rhei Radix et Rhizoma (Rhubarb)—a key metabolite of DCQD—significantly reduced procalcitonin, von Willebrand factor, prothrombin time, and APACHE II scores, while alleviating gastrointestinal dysfunction and increasing platelet counts (Zhang et al., 2015).

### Active metabolites of DCQD

DCQD is a classical prescription originating from the Treatise on Febrile Diseases (Shang Han Lun). The classical formula DCQD consists of three botanical drugs and one mineral drug. The botanical components were taxonomically validated using the Plants of the World Online (POWO) database. They are Rhei Radix et Rhizoma (*Rheum palmatum* L., Polygonaceae), Aurantii Fructus Immaturus (*Citrus × aurantium* L., Rutaceae), and Magnoliae Officinalis Cortex (*Magnolia officinalis* Rehder and E.H.Wilson, Magnoliaceae). The formula also includes Natrii Sulfas, a mineral drug primarily consisting of sodium sulfate decahydrate. DCQD is traditionally used in Chinese medicine for treating intestinal obstruction, constipation, and gastrointestinal dysfunction. Phytochemical analyses have identified that DCQD mainly contains flavonoids, carboxylic acids and derivatives, prenol lipids, and benzene and substituted derivatives (Shang et al., 2024). The principal bioactive metabolites include emodin, rheic acid, chrysophanic acid, magnolol, honokiol, hesperidin, naringin, neohesperidin, physcion, aloe-emodin, and tangeretin (Li et al., 2020; Pan et al., 2024). However, variations in herbal processing methods (Wei et al., 2018), decoction techniques and duration (Wan et al., 2013; Xiong, 2012), and dosage forms (Cao et al., 2014) can influence the qualitative and quantitative composition of these active metabolites. Network pharmacology studies have revealed that DCQD exerts multitarget and multipathway therapeutic effects in sepsis (Fu et al., 2021). Different active metabolites regulate distinct signaling pathways involved in the pathogenesis of sepsis. For instance, naringin exhibits anti-inflammatory and antioxidant effects in septic rat models (Bayram et al., 2023); aloe-emodin alleviates inflammation and modulates the gut microbiota to confer protection (Su et al., 2023); while emodin suppresses activation of the NLRP3 inflammasome by promoting FUNDC1-mediated mitophagy, thereby preventing sepsis progression (Fu et al., 2025). The molecular formulas and structures of the major active metabolites in DCQD are summarized in Table 1.

**TABLE 1:** Molecular formula and structure diagram of the main active metabolites in DCQD.

Metabolite name	Molecular formula	Structure diagram
Emodin	C_15_H_10_O_5_	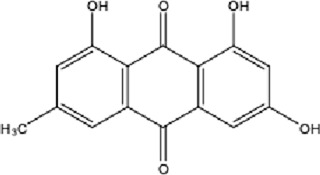
Rheic acid	C_15_H_8_O_6_	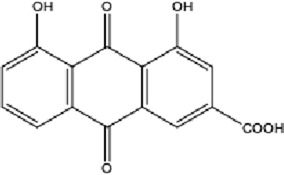
Chrysophanic acid	C_18_H_18_O_2_	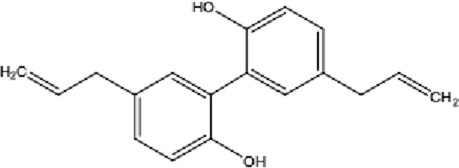
Magnolol	C_15_H_10_O_4_	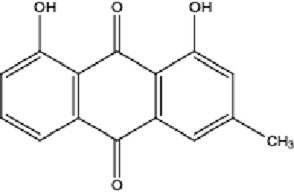
Honokiol	C_18_H_18_O_2_	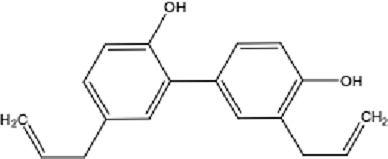
Hesperidin	C_28_H_34_O_15_	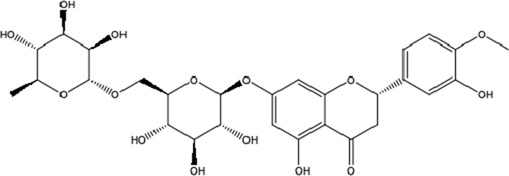
Naringin	C_27_H_32_O_14_	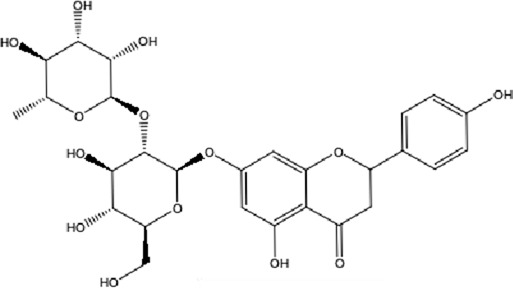
Neohesperidin	C_28_H_34_O_15_	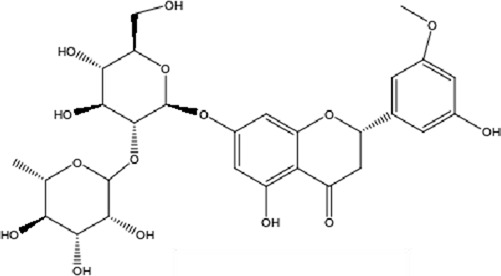
Physcion	C_16_H_12_O_5_	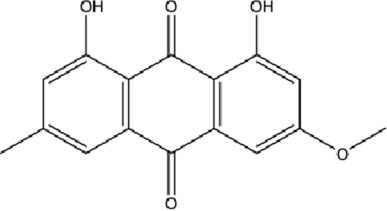
Aloe-emodin	C_15_H_10_O_5_	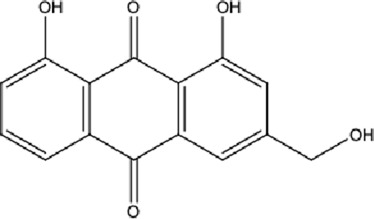
Tangeretin	C_20_H_20_O_7_	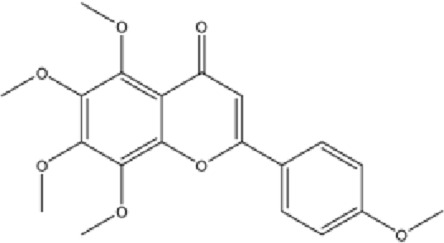

## Therapeutic potential of DCQD in sepsis-induced target organ injury

### Effects of DCQD on sepsis-associated gastrointestinal dysfunction

The gastrointestinal (GI) tract is among the most vulnerable organs affected during the course of sepsis (Yu et al., 2025). Intestinal barrier dysfunction, bacterial translocation, and gut microbiota imbalance are widely recognized as key contributors to the initiation and progression of intra-abdominal sepsis. The transition of intestinal homeostasis from cytokine storm to immunosuppression reflects the intricate interactions between microbial metabolites, immune dysregulation, and organ injury (Gong et al., 2025). Increasing evidence suggests that the gut communicates closely with other organs via the gut–lung, gut–brain, and gut–liver axes, forming a complex inter-organ regulatory network. TCM, including DCQD, has shown the ability to mitigate intestinal inflammation, restore microbial balance, enhance immunity, and preserve intestinal barrier integrity, thereby improving the clinical course of sepsis (Wang X. H. et al., 2022).

Clinical studies have reported that DCQD administration elevates plasma motilin levels, enhances gastrointestinal motility, alleviates gastrocardiac dysrhythmia, and reduces postoperative gastric paralysis (Qi et al., 2007; Xu et al., 2015). In a single-center, parallel-group, randomized controlled clinical trial, researchers randomly assigned 80 patients with sepsis complicated by gastrointestinal dysfunction to two groups in a 1:1 ratio. The control group received conventional Western medical treatment, while the observation group received conventional treatment plus oral DCQD. The dosage was 30 mL per dose, administered once in the morning and once in the evening. Bedside ultrasound was used to measure gastric antral cross-sectional area (CSA) and calculate gastric residual volume (GRV). Additionally, gastrointestinal dysfunction scores, intra-abdominal pressure (IAP), inflammatory markers (WBC, PCT, hs-CRP), nutritional indicators (PA, Alb), and APACHE II scores were assessed. Results demonstrated that DCQD significantly improved gastrointestinal dysfunction symptoms, reduced systemic inflammatory response, enhanced nutritional status, and shortened ICU length of stay (Li et al., 2023). Furthermore, DCQD effectively alleviates intestinal leakage, downregulates PV-1 expression, reduces hepatic translocation of Enterobacteriaceae, restores gut homeostasis, and limits lamina propria myeloid cell infiltration, partly through the FUT2/Wnt/β-catenin signaling pathway, thereby maintaining intestinal vascular barrier integrity (Wang S. H. et al., 2025). Combined therapy with acupuncture and DCQD produces synergistic benefits (Fei et al., 2024; Fei et al., 2025), promoting the recovery of gastrointestinal motility, reducing intestinal permeability, protecting the mucosal barrier, suppressing systemic inflammation, and improving biomarkers such as citrulline (Cit), intestinal fatty acid-binding protein (I-FABP), and diamine oxidase (DAO), ultimately inhibiting disease progression.

Mechanistically, rhubarb monomers (from Rhei Radix et Rhizoma) have been shown to enhance the transcription, translation, and expression of junctional proteins, thereby attenuating septic mucosal injury (Wang et al., 2017). Further studies revealed that emodin, a major anthraquinone metabolite of DCQD, enhances vitamin D receptor (VDR) expression and its downstream targets, inhibits inflammatory cytokine release and oxidative stress, and maintains intestinal integrity by modulating the gut microbiota in septic mice (Zhang et al., 2022). It also protects against sepsis-associated mucosal injury via the VDR/Nrf2/HO-1 signaling pathway (Shang L. et al., 2021). Meanwhile, magnolol exerts protective effects on sepsis-induced acute gastrointestinal injury by downregulating activation and secretion of T lymphocytes (Mao et al., 2021); and naringin protects against sepsis-induced intestinal injury by regulating macrophage polarization through the PPARγ/miR-21 axis and inhibiting MLC phosphorylation and NF-κB activation via the RhoA/ROCK pathway (Li et al., 2021).

### Effects of DCQD on sepsis-associated lung injury

DCQD is a representative prescription of the “lung–intestine combined treatment” principle in TCM and has shown remarkable potential in the treatment of acute lung injury (ALI). Lung injury is one of the most severe complications of sepsis, characterized by excessive inflammatory responses leading to damage and apoptosis of pulmonary capillary endothelial cells and alveolar epithelial cells (AECs). This disrupts the alveolar–capillary barrier, resulting in impaired gas exchange and pulmonary diffusion dysfunction (Sun et al., 2025). Epidemiological studies have reported that approximately 25%–50% of septic patients develop ALI, and the mortality rate is significantly higher compared with non-sepsis-related ALI cases (Bellani et al., 2016; Yoo et al., 2020). The pathogenesis of sepsis-induced ALI primarily involves dysregulated inflammation, oxidative–redox imbalance, and coagulation dysfunction (Sun et al., 2025).

Recent studies have demonstrated that DCQD alleviates ALI by suppressing excessive inflammation, inhibiting apoptosis (Pan et al., 2021a), attenuating PANoptosis (Zhang M. et al., 2025), and reducing HIF-1α–mediated glycolysis (Shang et al., 2024). *In vivo*, DCQD pretreatment improves dose-dependently the histopathological alterations of lung tissue in ALI mice, decreases the lung wet/dry weight (W/D) ratio, and reduces the total protein concentration and lactate dehydrogenase (LDH) levels in bronchoalveolar lavage fluid. *In vitro*, DCQD reverses lipopolysaccharide (LPS)-induced cytotoxicity, downregulates PANoptosis-related proteins ZBP1 and RIPK1, and protects against sepsis-related lung injury through the Nrf2/TGF-β1/ERK (Shang et al., 2021b) and TLR4/NF-κB pathways (Hu et al., 2019).

Mechanistic studies on DCQD’s active metabolites reveal that emodin regulates macrophage M1/M2 polarization via the VIP/cAMP/PKA pathway (Wang W. B. et al., 2022), suppresses NLRP3 inflammasome–dependent pyroptosis (Liu Y. et al., 2022), and alleviates lung injury by inhibiting SIRT1-mediated NF-κB and HMGB1 signaling (Liu F. J. et al., 2022). Hesperidin effectively suppresses the sepsis-induced cytokine storm (Jin et al., 2020), attenuates NLRP3 inflammasome–mediated pyroptosis through Nrf2 activation (Liu et al., 2021), and protects against septic lung injury via the Hsp70/TLR4/MyD88 pathway (Yuan et al., 2019). Furthermore, tangeretin not only inhibits macrophage ferroptosis (Zhang H. et al., 2025) but also mitigates sepsis-induced ALI by regulating the PLK1/AMPK/DRP1 signaling axis, thereby suppressing ROS-mediated NLRP3 inflammasome activation (Liu Y. et al., 2024).

### Effects of DCQD on sepsis-associated liver injury (SALI)

SALI is one of the most common organ dysfunctions in sepsis, characterized by multifactorial and multidimensional pathogenesis, including inflammatory responses, oxidative stress, multiple modes of cell death, microcirculatory disturbances, and gut–liver axis dysregulation (Guo et al., 2025; Xu et al., 2025a). Excessive inflammation, oxidative stress, and mitochondrial dysfunction lead to excessive reactive oxygen species (ROS) generation, which damages hepatocytes and activates the NLRP3 inflammasome, further aggravating hepatic sinusoidal endothelial injury, microcirculatory failure, ischemia–reperfusion damage, and energy metabolism imbalance. These pathological processes accelerate hepatocyte death through apoptosis, necroptosis, ferroptosis, and pyroptosis (Jaeschke and Ramachandran, 2024; Shi et al., 2023). Moreover, intestinal barrier dysfunction, bacterial translocation, and gut-liver axis disruption exacerbate liver injury by amplifying inflammation and endotoxin burden (Hao et al., 2024).

A 2025 network pharmacology study identified the core targets of DCQD and experimentally verified that, using *in vivo* SALI mouse models with normal control, model control, and DCQD-treated groups, DCQD alleviates SALI by activating the PI3K/Akt signaling pathway, thereby suppressing inflammation, oxidative stress, and apoptosis, while enhancing intestinal barrier integrity (Liu et al., 2025). In animal models of septic liver injury, DCQD markedly inhibited proinflammatory cytokines (TNF-α, IL-6) and oxidative stress markers (ROS, MDA) in hepatic tissue, improved liver function (ALT/AST) and histopathological architecture, reduced inflammatory infiltration, and increased antioxidant enzyme activity. Furthermore, DCQD was shown to attenuate hepatocyte apoptosis by inhibiting the TGF-β1/Smad3 pathway, thereby maintaining hepatic homeostasis (Pan et al., 2024). Clinically, DCQD enema therapy in elderly septic patients effectively reduced systemic inflammation, lowered mechanical ventilation rates and duration, and improved overall prognosis (Gong et al., 2024).

Among DCQD’s major active metabolites, emodin has been shown to exert hepatoprotective effects against LPS-induced acute liver injury by attenuating oxidative stress and hepatocyte apoptosis (Pan et al., 2021b). However, the protective mechanisms of other active metabolites in DCQD on SALI remain to be elucidated and warrant further investigation.

### Effects of DCQD on sepsis-associated kidney injury

The development of sepsis-associated acute kidney injury (SA-AKI) results from a complex interplay of multiple mechanisms, including microcirculatory dysfunction, immune inflammation, cell death, metabolic reprogramming, coagulation abnormalities, and epigenetic regulation (Liu A. B. et al., 2024; Fani et al., 2018; Pais et al., 2024; Wang et al., 2023). A comprehensive understanding of these mechanisms is essential for advancing early diagnosis and targeted therapeutic strategies.

Clinical studies have demonstrated that DCQD exerts renal protective effects in sepsis. The team led by Zeng Fankun (Zeng et al., 2025) reported that DCQD enema significantly attenuated systemic inflammation, improved renal function, and shortened ICU hospitalization time in septic patients. However, basic research on the mechanistic actions of the DCQD formula in sepsis-induced kidney injury remains limited and warrants further investigation.

In contrast, increasing evidence supports the role of individual bioactive metabolites of DCQD in mitigating sepsis-related renal injury. In an *in vivo* animal experiment, a rat CLP sepsis-induced acute kidney injury model was established, with normal control, model control, and honokiol administration groups. Renal pathological changes were observed via PAS staining, and key pharmacological indicators including oxidative stress, inflammatory factors, NO/iNOS, and NF-κB signaling pathways were detected. Honokiol has been shown to significantly improve survival rates, preserve renal histoarchitecture, and reduce oxidative stress and proinflammatory cytokine production in septic rats (Li et al., 2014; Xia et al., 2019). Moreover, Honokiol alleviates SA-AKI by downregulating nitric oxide (NO) and inducible nitric oxide synthase (iNOS) levels, suppressing NF-κB activation, and modulating the miR-218-5p/heme oxygenase-1 (HO-1) signaling pathway (Zhang and Xiang, 2019). Similarly, naringenin has been identified through network pharmacology analysis as targeting oxidative stress and apoptotic signaling cascades relevant to SA-AKI (Wang W. et al., 2025). *In vivo*, naringenin ameliorates renal histopathological injury, lowers serum creatinine and urea nitrogen levels, reduces inflammatory cytokine release, and downregulates Nrf2 and HO-1 protein expression in septic mice. In addition, emodin exerts nephroprotective effects against sepsis-induced AKI by modulating Nrf2 and AUF1 expression, thereby suppressing inflammation and enhancing antioxidant capacity (Ding et al., 2023). Chrysophanol, another anthraquinone metabolite in DCQD, mitigates mesangial cell injury and apoptosis by inhibiting the release of TNF-α and IL-6 and blocking the NF-κB signaling pathway (Gou et al., 2024).

### Potential protective effects of DCQD active metabolites on sepsis-associated myocardial injury

Sepsis-associated cardiac dysfunction (SACD) is a common and severe complication of sepsis, typically characterized by reversible ventricular dilation, reduced ejection fraction, and elevated cardiac troponin levels (Hollenberg and Singer, 2021). The underlying pathophysiological mechanisms are multifactorial and include: (1) an “inflammatory storm” triggered by pathogenic infection leading to excessive inflammatory activation and microcirculatory disturbance (L'Heureux et al., 2020); (2) mitochondrial dysfunction, calcium homeostasis imbalance, and sympathetic overactivation contributing to myocardial edema and decreased cardiac output (Hu et al., 2024; Meng et al., 2025); and (3) programmed cell death mechanisms such as apoptosis and pyroptosis that further exacerbate myocardial injury. Despite the potential of DCQD to target multi-organ dysfunction, the direct therapeutic effects of the DCQD formula on cardiac injury remain to be verified. No direct experimental or clinical studies have to date reported the effects of DCQD on sepsis-induced cardiac injury, which highlights this as a key area for future research.

Nonetheless, several bioactive metabolites of DCQD have shown cardioprotective effects in experimental models of sepsis. Naringin and honokiol have been demonstrated to improve myocardial dysfunction by inhibiting inflammatory responses, reducing cardiomyocyte apoptosis, and attenuating oxidative stress (Sun et al., 2021; Liu et al., 2023). Naringin, in particular, exerts its protective effects against LPS-induced myocardial dysfunction by activating the Keap1/Nrf2/HO-1 and PI3K/Akt/NF-κB signaling pathways (Sun et al., 2021; Xianchu et al., 2016; Sun et al., 2019). In addition, emodin mitigates LPS-induced cardiac injury by suppressing NLRP3 inflammasome activation, thereby reducing inflammation-mediated myocardial damage (Dai et al., 2021). Tangeretin, another important DCQD metabolite, protects against experimental sepsis-induced cardiac dysfunction through modulation of the PTEN/Akt/mTOR axis, effectively alleviating myocardial oxidative stress and improving cardiac contractility (Shiroorkar et al., 2020).

### Other related findings

In addition to the aforementioned organ-specific effects, several studies have identified broader protective actions of DCQD and its major active metabolites in sepsis. Emodin, one of the principal bioactive metabolites, has been shown to significantly suppress inflammatory responses in cecal ligation and puncture (CLP)-induced sepsis models. It inhibits hippocampal neuronal apoptosis, induces autophagy, and ameliorates cognitive dysfunction and neuropathological injury in septic mice by modulating the BDNF/TrkB signaling pathway, thereby markedly attenuating the progression of sepsis-associated encephalopathy (SAE) (Gao et al., 2022). Furthermore, emodin has been reported to alleviate sepsis-induced multiorgan damage (SIMD) by targeting neutrophil BCL-10, thereby suppressing NETosis and reducing the release of neutrophil extracellular traps (NETs) (Xu et al., 2025b). The detailed mechanisms underlying the effects of DCQD’s active metabolites on sepsis-induced organ injury are summarized in Table 2.

**TABLE 2:** Summary of the effects of active metabolites of DCQD on sepsis and related target organ injuries.

Metabolite name	Target organ in sepsis	Main pharmacological effects	Signaling pathways involved	References
Emodin	Sepsis	Promotes mitophagy	NLRP3	Fu et al. (2025)
	Gastrointestinal tract	Anti-inflammatory, antioxidant stress, maintains intestinal barrier integrity, regulates gut microbiota	VDR/Nrf2/HO-1	Zhang et al. (2022), Shang et al. (2021a)
	Lung	Regulates macrophage M1/M2 polarization	VIP/cAMP/PKA, NLRP3, NF-kB, and HMGB1	Wang W. B. et al. (2022), Liu Y. et al. (2022), Liu F. J. et al. (2022)
	Liver	Anti-oxidative stress, anti-apoptotic	-	Pan et al. (2021b)
	Kidney	Anti-inflammatory, antioxidant stress	Nrf2 and AUF1	Ding et al. (2023)
	Myocardium	Anti-inflammatory	NLRP3	Dai et al. (2021)
	Brain	Anti-inflammatory, anti-apoptotic, and induces autophagy	BDNF/TrkB	Gao et al. (2022)
	Multiple organs	Inhibits NETosis	BCL-10/MALT1 and NF-κB	Xu et al. (2025b)
Naringin	Sepsis	Anti-inflammatory, antioxidant stress	-	Bayram et al. (2023)
	Gastrointestinal tract	Regulates macrophage polarization	RhoA/ROCK	Li et al. (2021)
	Kidney	Anti-inflammatory, improves renal function	Nrf2 and HO-1	Wang W. et al. (2025)
	Myocardium	Anti-inflammatory, anti-apoptotic, antioxidant stress	Keap1/Nrf2/HO-1 and PI3K/AKT/NF-κB	Sun et al. (2021), Xianchu et al. (2016), Sun et al. (2019)
Tangeretin	Lung	Inhibits macrophage ferroptosis	PLK1/AMPK/DRP1 and NLRP3	Zhang H. et al. (2025), Liu A. B.et al. (2024)
	Myocardium	Inhibits cardiac autophagy	PTEN/AKT/mTOR and AKT/mTOR	Shiroorkar et al. (2020)
Honokiol	Kidney	Anti-inflammatory, antioxidant stress, reduces NO and iNOS production	NF-κB and miR-218-5p/HO-1	Li et al. (2014), Xia et al. (2019), Zhang and Xiang (2019)
	Myocardium	Anti-inflammatory, anti-apoptotic, antioxidant stress	-	Liu et al. (2023)
Hesperidin	Lung	Anti-inflammatory, anti-pyroptotic	Hsp70/TLR4/MyD88 and NLRP3	Jin et al. (2020), Liu et al. (2021), Yuan et al. (2019)
Aloe-emodin	Sepsis	Anti-inflammatory, regulates gut microbiota	-	Su et al. (2023)
Chrysophanic acid	Kidney	Anti-inflammatory, anti-apoptotic	NF-κB	Gou et al. (2024)
Magnolol	Gastrointestinal tract	Regulates T-cell activation	LPS/NF-κB and RANTES	Mao et al. (2021)

## Safety evaluation of DCQD in the treatment of sepsis

In clinical practice, no severe adverse reactions associated with DCQD have been reported in the treatment of sepsis to date. In the aforementioned clinical trials involving patients with sepsis complicated by gastrointestinal dysfunction, no serious DCQD-related adverse events were observed, suggesting that the decoction demonstrates a favorable safety profile within the studied dosage range and treatment duration. However, given its purgative properties, caution should be exercised when administering DCQD to patients with frail constitutions or preexisting intestinal disorders, as such individuals may be more susceptible to gastrointestinal irritation or excessive purgation. Therefore, close monitoring of bowel function and general condition is recommended during treatment to ensure safe clinical use.

## Discussion

Due to the complex pathophysiology of sepsis, current Western medical treatments remain largely supportive and have not yet resolved the fundamental mechanisms of the disease. In contrast, TCM metabolite formulas, composed of multiple bioactive metabolites, have demonstrated distinct advantages and therapeutic potential in managing sepsis and its associated organ injuries (Ji et al., 2025). Unlike single-metabolite pharmacotherapy, TCM formulas act as synergistic systems, where herbal compatibility and pharmacodynamic interactions between individual metabolites enhance overall therapeutic efficacy rather than simply summing the effects of single agents (Zhao et al., 2025). Notably, the multi-metabolite synergistic effects of DCQD for sepsis, the core advantage of this classic TCM formula, are further emphasized here. Based on existing network pharmacology and preclinical evidence, its four metabolite botanical drugs (Rhei Radix et Rhizoma, Natrii Sulfas, Aurantii Fructus Immaturus, Magnoliae Officinalis Cortex) exert complementary regulatory effects on sepsis-related pathological processes: Rhei Radix et Rhizoma (monarch botanical drug) exerts core anti-inflammatory and intestinal barrier-repairing effects; Natrii Sulfas (minister botanical drug) potentiates its purgative and intestinal motility-promoting effects; Aurantii Fructus Immaturus and Magnoliae Officinalis Cortex (assistant and guide botanical drugs) regulate gastrointestinal dynamics and intestinal inflammation, complementing the systemic organ-protective effects of the monarch and minister botanical drugs. Together, they form a synergistic regulatory network targeting intestinal homeostasis and systemic inflammation. Importantly, in-depth mechanistic research on DCQD’s compatibility remains a critical gap, such as experimental verification of the superior efficacy of the combined formula over single botanical drugs/metabolites. Thus, elucidating the synergistic/antagonistic mechanisms of DCQD’s metabolites and optimizing its metabolite ratio are identified as key directions for future in-depth research, which will lay a solid foundation for its standardized clinical application in sepsis. DCQD, as a classical multi-metabolite prescription, represents a promising therapeutic strategy for sepsis and related organ dysfunction. It not only attenuates the early hyperinflammatory phase by suppressing cytokine storms but also modulates immune responses during the immunosuppressive phase, thereby restoring immune homeostasis. Furthermore, DCQD exerts multi-organ protection in sepsis via a conserved core network centered on the inflammation-oxidative stress-cell death axis, with universal modulation of key signaling pathways across organs: it broadly inhibits the NF-κB pathway to suppress excessive inflammation and activates the Nrf2/HO-1 pathway to alleviate oxidative stress, collectively inhibiting abnormal cell death in damaged tissues. Meanwhile, DCQD exerts organ-specific effects based on distinct pathological features: it targets the FUT2/Wnt/β-catenin pathway to repair intestinal barrier integrity; modulates the TGF-β1/ERK and PLK1/AMPK/DRP1 pathways to protect alveolar-capillary barrier and inhibit macrophage ferroptosis in the lung; and activates the PI3K/Akt pathway to mitigate parenchymal cell injury in the liver and kidney. This integration of universal and organ-specific mechanisms underpins DCQD’s multi-target efficacy in sepsis-induced multi-organ dysfunction, reflecting the holistic regulatory characteristic of TCM formulas. However, several important limitations remain in both clinical and experimental research on DCQD. First, the current level of clinical evidence is relatively low, with a lack of multicenter, large-sample randomized controlled trials (RCTs) to robustly verify its efficacy and safety. Second, the pharmacodynamic interactions and compatibility mechanisms among its metabolite botanical drugs are not yet fully elucidated. Third, differences in efficacy among various routes of administration remain unclear and require further investigation. Fourth, most evidence supporting the efficacy of DCQD is derived from preclinical studies, including *in vitro* experiments, animal models, and network pharmacology analyses, accounting for approximately 70% of the cited literature. By contrast, clinical evidence constitutes only about 30%, and most clinical studies are single-center, non-randomized, or observational. Fifth, although this review has summarized several key signaling pathways including VDR/Nrf2/HO-1 and PLK1/AMPK/DRP1, the crosstalk and hierarchical regulatory relationships among these pathways remain unclear, and studies on their interactions under DCQD intervention are still rare.

However, this paper is a narrative review aimed at synthesizing and summarizing the mechanisms of action and overall efficacy of DCQD in sepsis and its associated multi-organ injury. Following the standard structure and academic norms for narrative reviews, we focus on summarizing consistent conclusions regarding DCQD’s effects on sepsis-related organ dysfunction, key signaling pathways, and overall efficacy. And we have supplemented core pharmacological information for key and representative studies.But it is not a systematic review/meta-analysis requiring complete data extraction and statistical analysis of every individual study.

This review covers multiple experimental models, such as the cecal ligation and puncture (CLP) model, lipopolysaccharide (LPS)-induced model, and LPS-induced organ damage. The CLP model closely aligns with the traditional pharmacological actions of Da Cheng Qi Tang (DCQD)-promoting bowel movement to clear heat, protecting the intestinal barrier, and blocking the gut-organ axis-by mimicking clinical peritoneal infection, enteric bacterial translocation-induced sepsis, and multiple organ dysfunction. (1) Definition: The CLP model is a standard polymicrobial sepsis model. It mimics clinical abdominal infection and multiple organ dysfunction by releasing intestinal contents into the peritoneal cavity via cecal ligation and puncture. The LPS-induced model is a classic endotoxemia model. It triggers acute systemic inflammation by activating inflammatory signaling pathways through exogenous LPS administration. The LPS-induced organ damage model is a targeted injury model. It causes specific structural and functional impairment in organs such as the lung, liver, kidney and heart for studying organ protection. (2) Relevance to research: The LPS model induces severe systemic inflammatory responses by activating pathways such as TLR4/NF-κB, serving as a classic tool for studying DCQD’s anti-inflammatory, antioxidant, and signaling pathway inhibitory effects. LPS-induced damage to organs including the kidneys, liver, lungs, intestines, and heart allows targeted evaluation of DCQD’s protective effects against sepsis-related multi-organ injury, fully aligning with the organ targets of this review. (3) Model Validity in Research: The CLP model most closely mimics the pathophysiological process of clinical sepsis, reliably replicating intestinal barrier disruption, bacterial/endotoxin translocation, and multi-organ injury. It is suitable for evaluating the holistic efficacy of DCQD in “clearing the bowels, protecting the gut, and mitigating distant organ injury.” The LPS model is simple to establish, highly reproducible, and induces intense inflammatory responses, making it suitable for rapid screening of DCQD’s anti-inflammatory and antioxidant effects. It can clarify DCQD’s regulatory effects on key pathways such as TLR4/NF-κB, PI3K/AKT, and MAPK. The LPS-induced organ injury model can establish damage models in tissues/cells including kidney, liver, lung, intestine, and myocardium, facilitating the analysis of DCQD’s organ-specific protective mechanisms. This supports multi-level validation of DCQD’s action targets from whole animal→tissue→cell→molecular levels. (4) Limitations: CLP model surgical procedures exhibit significant variability, low standardization, and limited reproducibility between models. The complexity of infection and inflammation makes it challenging to precisely distinguish whether DCQD exerts its effects through antibacterial, anti-inflammatory, intestinal barrier protection, or a combination of multiple pathways. The LPS model represents pure endotoxin stimulation, lacking key clinical sepsis factors such as bacteria, tissue ischemia, trauma, and immune paralysis. It primarily induces excessive inflammation and cannot fully mimic the biphasic immune changes observed in clinical sepsis. LPS-induced organ injury models predominantly feature acute, single-insult mechanisms, diverging from the multifactorial, sequential organ damage observed clinically. *In vitro* cellular models cannot reflect the systemic effects of DCQD *in vivo*, including absorption, metabolism, gut-organ axis interactions, and neuroendocrine regulation. (5) Future Research Needs and Priorities: First, establish standardized operating procedures (SOPs) for CLP and LPS models, standardizing modeling intensity, drug dosages, administration routes, and observation timepoints. Second, develop composite models combining CLP/LPS with ischemia-reperfusion injury or gut injury superimposed on distant organ damage to more accurately mimic the pathological progression of clinical sepsis. Finally, determine the effective dose range, optimal timing, and treatment duration for DCQD, while conducting target validation through network pharmacology and molecular docking.

DCQD is one of the classic formulas in traditional Chinese medicine, originating from the Treatise on Cold Damage. It possesses the efficacy of vigorously purging heat accumulation and is primarily used to treat Yangming Fulfillment Syndrome (characterized by internal accumulation of real heat and dry heat in the stomach and intestines, manifesting as constipation, abdominal pain, etc.). Its core ingredients include Rhubarb, Magnolia Bark, Bitter Orange Fruit, and Epsom Salt. Through purgative action to relieve constipation, promoting qi circulation to eliminate stagnation, it clears dry feces and accumulated heat from the intestines. Other multi-component traditional Chinese medicine formulations studied in sepsis include: Xuebijing Injection, Qingkailing Injection, An Gong Niu Huang Wan, Zixue San, Shenfu Injection, and Reduning Injection.

Conceptually compare the aforementioned Chinese herbal formula with Dachengqi Decoction. DCQD is a classic Chinese herbal decoction formula belonging to the “lowering method” category in Traditional Chinese Medicine. Its core pathogenesis involves Yangming viscera obstruction, gastrointestinal heat accumulation, and impaired viscera qi flow. Representative symptoms include abdominal distension, constipation, high fever, and abdominal fullness with hardness and pain. In sepsis, it primarily functions by unblocking the viscera to clear heat, promoting intestinal detoxification, protecting the intestinal barrier, and reducing enteric endotoxin translocation. In contrast, the other herbal formulations mentioned are predominantly modern herbal injections or proprietary Chinese medicines for opening orifices, classified under the methods of clearing heat, opening orifices, consolidating collapse, and promoting blood circulation, respectively. Their core pathogenesis involves intense heat-toxin, heat invading the pericardium, collapse of vital energy, and mutual entanglement of stasis and toxins. Representative syndromes manifest as high fever, mental confusion, shock, and multiple organ dysfunction. In sepsis, these therapies primarily exert therapeutic effects through pathways such as systemic anti-inflammatory action, anti-shock effects, vascular endothelial protection, brain-awakening and orifice-opening, and vital organ protection.

Mechanistically, DCQD targets the gut as its primary focus. By promoting gastrointestinal motility, protecting the intestinal mucosal barrier, reducing enteric endotoxin translocation, regulating gut microbiota, and inhibiting gut-related inflammatory pathways, it achieves the effects of “clearing heat from the bowels and urgently purging to preserve yin.” This approach controls the gut-derived inflammatory response in sepsis at its source. The remaining drugs primarily act on the systemic inflammatory network, vascular endothelium, circulatory system, and central nervous system. They exert their effects through pathways such as suppressing systemic inflammatory storms, protecting vascular endothelium, counteracting shock, clearing heat and opening orifices, and safeguarding the blood-brain barrier.

Based on current evidence, DCQD is defined by nearly all high-quality clinical trials and guidelines as an adjunctive intervention combined with conventional Western therapies (including antimicrobial treatment, fluid resuscitation, and organ support). Furthermore, DCQD cannot replace core Western medical treatments; it improves pathophysiological states solely through mechanisms such as modulating inflammation, protecting the intestinal barrier, counteracting shock, and promoting cerebral arousal. DCQD as an adjunctive strategy that can be integrated into multimodal care.

As we noted, (1) patients with septic shock typically demonstrate the most pronounced improvement. This is because such patients present with severe systemic inflammation, microcirculatory impairment, and inadequate tissue perfusion. DCQD’s mechanisms—anti-inflammatory effects, circulatory enhancement, endothelial protection, and anti-shock action—are highly aligned with these pathologies, leading to greater relative therapeutic gains. (2) Patients with multiple organ failure show minimal or negligible benefit. For instance, those with frail constitutions or pre-existing severe intestinal diseases may experience gastrointestinal irritation or exacerbation of primary intestinal pathology due to DCQD’s laxative properties. Since prognosis is primarily determined by underlying damage, organs in these patients have already entered severe injury/failure stages where damage is irreversible. Supportive therapy can only modulate pathological disturbances and cannot reverse organ failure, thus yielding limited therapeutic gains. (3) Elderly patients show moderate to weak benefits with significant individual variation. This group often has multiple comorbidities, poor reserve capacity, and immune aging, resulting in reduced capacity to repair inflammation and organ damage. However, some elderly sepsis patients can still achieve inflammation reduction and organ function improvement through supportive therapy, indicating potential benefits.

Based on the clinical potential of DCQD and the limitations of existing research, future studies aiming to promote the standardization and precision of DCQD in the treatment of sepsis should focus on the following aspects. First, the in-depth development of DCQD requires high-level clinical evidence; therefore, subsequent multicenter, randomized, double-blind, placebo-controlled trials (RCTs) are particularly important in the development of DCQD (Du et al., 2025). For future clinical research design, scientific sample size estimation should be conducted based on preliminary efficacy data of DCQD in sepsis. Comprehensive and objective outcome measures are recommended, including APACHE II scores for assessing disease severity, organ-specific functional biomarkers (e.g., Cit, I-FABP for gastrointestinal injury; ALT/AST for liver injury) for evaluating target organ protection efficacy, and core hard endpoints such as 28-day all-cause mortality and incidence of multiple organ dysfunction syndrome. More importantly, multicenter, double-blind, placebo-controlled randomized controlled trials in line with international clinical research norms should be designed, and research populations should be stratified according to sepsis phenotypes (e.g., with/without gastrointestinal dysfunction, elderly/non-elderly) to improve the clinical translational value and evidence-based medical level of research results. Second, it is necessary to innovate in the study of administration routes and compare the pharmacokinetic characteristics of different administration methods, such as oral, nasal feeding, and enema, so as to determine the optimal and most stable administration scheme. Finally, modern systems biology tools such as metabolomics, proteomics, and single-cell sequencing should be applied to conduct in-depth and multi-dimensional research on DCQD, to reveal the synergistic, antagonistic, and potentiating mechanisms among the various herbal metabolite metabolites of the formula. This will provide scientific support for the modernization of TCM and continuously explore molecular biomarkers of the “Yangmingfushi” syndrome in sepsis patients, thereby achieving the organic integration of TCM syndrome differentiation and modern diagnostic techniques. More precisely (1) Omics technologies (genomics, transcriptomics, proteomics, metabolomics, etc.) enable comprehensive screening of differentially expressed genes, proteins, metabolites, and signaling pathways following DCQD intervention in sepsis. They provide a molecular-level panorama of key molecular changes regulating intestinal barrier function, inflammatory responses, immune function, and organ injury, offering objective, high-throughput experimental evidence for its multi-target effects. (2) Network pharmacology constructs interaction networks linking “drug–active ingredient–core target–disease pathway” to systematically elucidate the intrinsic mechanisms of DCQD’s multi-component, multi-target, and multi-pathway synergistic effects, visually demonstrating its systemic impact on multiple pathophysiological stages of sepsis. (3) Artificial intelligence modeling integrates multi-omics, network, and clinical data through machine learning and deep learning algorithms to precisely identify DCQD’s key effect targets, advantageous beneficiary subgroups, and dose-response relationships. This enables quantitative assessment, prediction, and precise interpretation of its multi-target effects. Collectively, these modern approaches translate DCQD’s multi-target effects into a systematic, data-driven mechanism, laying the scientific foundation for standardizing, precision-targeting, and advancing DCQD’s translational development in sepsis treatment.

In conclusion, DCQD provides a TCM-based therapeutic approach for the treatment of critical illness. The summary of both the DCQD metabolite and its single metabolites also opens up a new direction for the development of novel drugs targeting sepsis and its related organ injuries.
